# RETRACTION: Effects of Laparoscopic Splenectomy on Surgical Site Wound Infection in Patients With Spleen Rupture: A Meta‐Analysis

**DOI:** 10.1111/iwj.70273

**Published:** 2025-03-03

## Abstract

**RETRACTION:** DengY. , ZhangX. , LiA. , ZhaoY. , and YeL. , “Effects of Laparoscopic Splenectomy on Surgical Site Wound Infection in Patients With Spleen Rupture: A Meta‐Analysis,” International Wound Journal 21, no. 2 (2024): e14440, 10.1111/iwj.14440.PMC1082812137872696

The above article, published online on 23 October 2023, in Wiley Online Library (http://onlinelibrary.wiley.com/), has been retracted by agreement between the journal Editor in Chief, Professor Keith Harding; and John Wiley & Sons Ltd. Following an investigation by the publisher, all parties have concluded that this article was accepted solely on the basis of a compromised peer review process. The editors have therefore decided to retract the article. The authors did not respond to our notice regarding the retraction.

**RETRACTION:** Y. Deng , X. Zhang , A. Li , Y. Zhao , and L. Ye , “Effects of Laparoscopic Splenectomy on Surgical Site Wound Infection in Patients With Spleen Rupture: A Meta‐Analysis,” International Wound Journal 21, no. 2 (2024): e14440, 10.1111/iwj.14440.PMC1082812137872696

The above article, published online on 23 October 2023, in Wiley Online Library (http://onlinelibrary.wiley.com/), has been retracted by agreement between the journal Editor in Chief, Professor Keith Harding; and John Wiley & Sons Ltd. Following an investigation by the publisher, all parties have concluded that this article was accepted solely on the basis of a compromised peer review process. The editors have therefore decided to retract the article. The authors did not respond to our notice regarding the retraction.

